# Electronic Nanodevices

**DOI:** 10.3390/nano12132125

**Published:** 2022-06-21

**Authors:** Antonio Di Bartolomeo

**Affiliations:** Department of Physics “E.R. Caianiello”, University of Salerno, 84084 Fisciano, SA, Italy; adibartolomeo@unisa.it; Tel.: +39-089-969189

The new phenomena observed in nanodevices and the related technological challenges of fabrication and manipulation at the nanoscale have spurred intense theoretical, simulation and experimental research activity. New device structures, materials, simulation and characterization techniques have emerged.

The Special Issue entitled “Electronic Nanodevices” focuses on the design, simulation, fabrication, and modeling of new nanodevices for electronic, optoelectronic and energy applications; it includes articles dealing with nanoscale transistors, phototransistors, memories, and solar cells.

The effects of down-scaling and the introduction of new materials, architectures and fabrication processes in transistors and phototransistors are widely investigated.

High-electron-mobility transistors (HEMTs) are devices designed for high-frequency and high-power applications. An experimental study by P. Cui and Y. Zheng checked the impact of vertical and lateral scaling on low-field electron mobility in GaN HEMTs [1]. Although low-field mobility is expected to stay constant when devices scale down, in GaN HEMTs, the distribution of polarization charges that scatter with the channel electrons, localized in the barrier layer, can change with the device dimensions, thus leading to mobility variations. Indeed, P. Cui and Y. Zheng demonstrate that in InAlN/GaN HEMTs, the mobility decreases as the InAlN barrier and the gate length scale down but increases with the down-scaled source–drain distance. Their study highlights that the polarization charges are an important ingredient to consider in the nanoscale device design.

Owing to their great mechanical, electronic, and carrier transport properties, two-dimensional (2D) materials such as graphene, black phosphorus and transition metal dichalcogenides [2,3,4,5,6], as well as one-dimensional (1D) carbon nanotubes [7,8], are considered promising candidates for future post-silicon electronic and optoelectronic devices. Their use as channel of field effect transistors (FET) has been an important part of the down-scaling process.

M. La Mura and coworkers numerically investigated the effects of device geometry and graphene quality variations on the performance of graphene-field effect transistors (GFETs) [9]. The poor repeatability of GFETs hampers their diffusion in GFET-based commercial RF circuits. Specifically, the impact of geometrical parameters on the RF performance of a GFET-based common-source amplifier is studied in terms of the amplifier transit frequency and maximum oscillation frequency. M. La Mura and coworkers concluded that the most influential factor variation on the transition frequency and the maximum oscillation frequency is the channel length, as expected, because the transistor high-frequency limit is inversely proportional to the time the carriers need to cross the channel. They show that reducing the channel length increases the transition and the maximum frequency and point out that the accurate control the channel length is essential to reducing unwanted fluctuations of the transistors’ cut-off frequency. They also highlight that the improvement provided by increasing the accuracy of the other geometrical parameters, such as channel width or top oxide thickness, is very limited.

M. Poljak and coworkers dealt with the contact resistance in black phosphorus nanoribbons (PNRs) with edge contacts using atomistic quantum transport simulations [10]. The acceptable bandgap and the high carrier mobility of monolayer black phosphorus (or phosphorene) make it more advantageous than other 2D materials for nanoscale FETs. The impact of PNR size down-scaling on the contact resistance is analyzed for technologically relevant PNR widths and lengths. It is shown that the contact resistance decreases with the width down-scaling but increases considerably when the length decreases. Significant metallization effects become visible in the deterioration of electronic and transport properties for nanoribbon lengths below ~8 nm. It is pointed out that for optimized metal edge contacts, a weakly interacting metal is best suited to ultra-short nanoribbons. The numerical results indicate that in ultra-narrow PNR devices, the quantum intrinsic limits of the contact resistance, i.e., minimum achievable contact resistance, could be as low as ~14 Ω μm, a level acceptable to the CMOS industry.

E. Faella et al. fabricated back-gated FETs based on few-layer ReSe_2_ with Cr contacts and presented their optoelectronic characterization [11]. The devices show n-type conduction due to the to the alignment of the Cr Fermi level with the ReSe_2_ conduction band. It is demonstrated that the ReSe_2_ FETs are strongly affected by air pressure and undergo a dramatic increase in conductivity when the air pressure is lowered below the atmospheric pressure. The exposure to air suppresses the channel conductivity as an effect of electron capture by oxygen and water molecules adsorbed on the material surface. The reversible pressure behavior allows the devices to be used as air pressure gauges. Finally, a negative photoconductivity in the ReSe_2_ channel is found and explained as back-gate-dependent trapping of the photo-excited charges.

K. Tamersit and coworkers performed numerical simulations by self-consistently solving the Poisson equation with the mode space non-equilibrium Green’s function formalism in the ballistic limit to investigate carbon nanotube/nanoribbon junctionless phototransistors, endowed with sub-10 nm photogate lengths [12]. The light-induced modulation of electrostatics through the photogate is employed as a photosensing principle and the impact of the light illumination on the transport of carbon-based junctionless phototransistors is analyzed via the energy-position-resolved electron density. They find that the junctionless approach is efficient in boosting the photosensitivity of phototransistors by dilating the potential barrier while mitigating the tunneling currents and improving the subthreshold characteristics. They also compare graphene nanoribbon and carbon nanotube junctionless phototransistors, concluding that the former exhibit higher photosensitivity.

The same group in another computational work proposed an efficient approach based on the synergy of electrostatic and chemical-doping engineering to boost the subthreshold and switching performance of sub–10 nm junctionless-carbon-nanotube-tunnel field-effect transistors [13]. Their doping approach, also favored by ferroelectric-based gating, is exploited to shrink the band-to-band tunneling window and dilate the direct source-to-drain tunneling window.

Ferroelectric gates are also considered by M. Takahashi and S. Sakai who fabricated a new strontium bismuth tantalate (SBT) ferroelectric-gate FET with channel lengths of 85 nm by a replacement-gate process [14]. Their device is demonstrated to be suitable for non-volatile memories with long stable data retention of $10^{5}$ s and high erase-and-program endurance up to $10^{9}$ cycles. In the fabrication process, they prepared dummy-gate transistor patterns and then replaced the dummy substances with an SBT precursor which is subsequently annealed for SBT crystallization. The proposed process has good channel-area scalability in geometry depending on the lithography ability.

Memory devices are also treated in a review paper by Juan B. Roldán and coworkers, dedicated to resistive random access memories (RRAMs) [15]. RRAMs are based on resistive switching mechanisms to modulate their conductance in a non-volatile manner and exhibit a set of technological features that make them ideal candidates for applications related to non-volatile memories, hardware cryptography, and neuromorphic computing. The review is focused on RRAM models dealing with temperature effects, which are very important considering that the physical mechanisms behind resistive switching are thermally activated. The authors describe models of different complexity to integrate thermal effects in complete compact models that account for the kinetics of the chemical reactions behind resistive switching and the current calculation. Specifically, among other issues, they treat different geometries, operation regimes, lateral heat losses, etc. to characterize each conductive filament.

Driven by the ever-increasing need for sustainable energy sources, the design, fabrication, and characterization of solar cells have also attracted a great deal of research endeavor.

S. Bernardes and coworkers numerically studied the lattice-matched GaInP/GaInAs/Ge triple-junction solar cell, which is currently being used in most satellites and concentrator photovoltaic systems [16]. They first analyzed the three subcells individually and then they simulated the whole cell by extracting the typical figures-of-merit. They compared the simulated results with the actual experimental results, confirming that the cell is emulated successfully. After that, they investigated the effect of temperature, which is relevant for space applications, and proceeded with the optimization of the cell, in terms of thickness and doping, so that the maximum efficiency can be reached. As an important guideline for fabrication, they also highlighted how the doping can significantly boost the cell efficiency.

F. Duarte and coworkers used COMSOL Multiphysics^®^ software to study nanometric optical antennas, with dimensions smaller than the wavelength of the incident electromagnetic wave, for solar energy harvesting on photovoltaic cells [17]. The use of optical antennas has received significant interest as they represent a viable alternative to the traditional energy harvesting technologies. To increase the efficiency of solar cells, the behavior of optical aperture nanoantennas, which consist of a metal sheet with apertures of dimensions smaller than the wavelength, is studied for materials such as aluminum, gold, and platinum. With several simulations in different conditions, F. Duarte and coworkers showed that all three metals exhibit the optical transmission phenomenon, i.e., they transmit a greater amount of light than might naively be expected. The enhanced transmission is due to the coupling of light with surface plasmon polaritons on the surface of the metallic nanoantennas. Furthermore, it is shown that aluminum is superior to the other materials because of its transmission and reflection coefficients.

Efficient solar cells can also be achieved through the development of innovative materials. Among them, two-dimensional metal MXenes, consisting of transition-metal nitrides or carbides, have emerged for their outstanding transparency, metallic electrical conductivity, and mechanical characteristics. Recent applications of MXene materials in solar cells and new perspectives to achieve higher power conversion efficiency with an excellent quality–cost ratio are offered in a review paper by T. F. Alhamada and coworkers [18]. The review details the basic principles for the creation of each 2D transition-metal MXene structure, the tunable characteristics depending on the transition-metal composition and summarizes all previously reported work on incorporating MXene into solar cells. The roles that MXenes play to improve solar power generation, operational stability and power conversion efficiency are reviewed.

Efficient energy storage is another important aspect of the sustainable development. Currently, lithium-ion batteries (LIBs) have become one of the most important energy storage technologies and their progress requires the development of high-capacity electrode materials [19]. Molybdenum oxides such as MoO_2_ and MoO_3_ are considered as promising anode materials for LIBs due to the broad spectrum of electrical properties ranging from metallic (MoO_2_) to wide band gap semiconducting (MoO_3_) character. In this direction, H. Wang and coworkers used orange peel extract as an effective chelating agent to synthesize molybdenum oxides [20]. MoO_2_ and MoO_3_ are prepared in vacuum and in air, respectively, at a temperature of 450 °C. Extensive morphological and structural characterization along with electrical and electrochemical analysis are performed to show their outstanding properties in terms of capacity upgrading upon cycling and as an anode in material for Li-ion batteries.

In summary this Special Issue offers a good overview of the recent research in nanoscale transistors, based on 1D and 2D materials, solar cells, memory devices, and electrochemical devices.

## Data Availability

Data might be available from the Authors of the cited papers.
